# Association between drinking behaviors, sleep duration, and depressive symptoms

**DOI:** 10.1038/s41598-024-56625-x

**Published:** 2024-03-12

**Authors:** Yujin Kim, Jihye Kim, Jae Won Oh, San Lee

**Affiliations:** 1 Department of Psychiatry, Yongin Severance Hospital, Yongin, Republic of Korea; 2 Department of Social Welfare, Seoul National University, Seoul, Republic of Korea; 3 Department of Psychology, University of Utah Asia Campus, Incheon, Republic of Korea; 4 Department of Psychiatry, Institute of Behavioral Science in Medicine, Yonsei University College of Medicine, Seoul, Republic of Korea

**Keywords:** Depressive symptoms, Drinking behaviors, Sleep duration, KNHANES, PHQ-9, Psychology, Human behaviour

## Abstract

Excessive alcohol consumption has been consistently linked to depression. This study, utilizing nationwide samples from the Korea National Health and Nutrition Examination Survey (n = 21,440) examined the association between drinking behaviors and depressive symptoms while also exploring the influence of sleep duration on this relationship. Demographic, socioeconomic, and health-related factors were included as covariates in the multivariable logistic regression analysis to assess their relationships with depressive symptoms. Based on their sleep duration, the participants were divided into subgroups to explore how sleep duration affects the relationship between drinking behaviors and depressive symptoms. Moderate alcohol consumption (1–4 times a month) was associated with reduced likelihood of experiencing depressive symptoms in women (p = 0.024), with a similar trend observed among men (p = 0.001). Men who started consuming alcohol before the age of 19 had a higher likelihood of experiencing depressive symptoms (p = 0.048). Only women who consumed more alcohol per occasion (≥ 7 drinks) had higher odds of depressive symptoms (p = 0.001). This study revealed complex factors that influence depressive symptoms, including alcohol consumption and sleep duration. This highlights the importance of tailored interventions based on sleep duration and sociodemographic characteristics for preventing and treating depression.

## Introduction

Depression, a pervasive and complex mental health disorder, has far-reaching implications for both individuals and society at large. Its impact extends beyond individual well-being, affecting emotional and physical health^1,2^. Depression can compromise an individual's quality of life, leading to diminished overall well-being^3^. Reduced social activities stemming from depression can exacerbate social disparities and impose various social and economic burdens^4,5^. The consequences of depression are not confined to the inner turmoil experienced by those affected but permeate various aspects of life. This reduction in the quality of life can manifest in various ways, affecting physical health, emotional stability, and overall life satisfaction. Consequently, addressing depression is not merely an individual concern but a collective societal responsibility.

Alcohol consumption is a predominant and influential factor that contributes substantially to depression. A well-established link exists between depression and recurrent alcohol consumption. Excessive alcohol use tends to exacerbate depressive symptoms and hinder recovery. Excessive alcohol intake and frequent and heavy episodic drinking have been associated with depression^6,7^. The interplay between alcohol consumption and depression reveals an intricate relationship between mental health and substance use. Expanding on the established body of research on the association between alcohol consumption and depression^8^, it was found that individuals engaging in hazardous drinking exhibited a higher likelihood of developing depression than those adhering to non-hazardous alcohol consumption practices. Additionally, their research sheds light on the complexity of this relationship by revealing that individuals who abstained from alcohol had a greater risk of depression than light drinkers. This nuanced perspective challenges the notion that the mere act of drinking immediately impacts depression; instead, it underscores the importance of the quantity and frequency of alcohol consumption in this context.

Inadequate sleep is associated with various mental health issues, including stress, depression, and anxiety. Irregular sleep duration can contribute to the onset and exacerbation of mental health challenges^9,10^. Emphasizing the bidirectional influence, Johnson and Breslau (2001) highlighted that alcohol consumption can influence sleep duration^11^, making it essential to consider this interaction in the context of psychiatric problems. Furthermore, a study examining the association between alcohol consumption and sleep found that the impact of alcohol on sleep was most pronounced during episodes of binge drinking and periods of acute withdrawal^12^. However, Koob and Colrain (2020), mentioned that while alcohol may have some initial impact on sleep onset, it is not considered helpful for overall sleep quality, especially in the context of Alcohol Use Disorder (AUD) where it is associated with various sleep disturbances across different stages of the addiction cycle^13^. Considering abundant evidence indicating bidirectional relationships between sleep, alcohol, and depression, our incorporation of sleep duration into the analysis aims to capture the multifaceted dynamics inherent in these interrelated factors.

Although previous research has often highlighted the link between alcohol consumption and depression, existing studies have not analyzed the influence of sleep duration on a representative sample of participants. Therefore, this study aims to delve deeper into the intricate relationships between these variables. Specifically, we sought to investigate whether the effects of alcohol consumption on depression vary depending on sleep duration and to explore the complex interplay between drinking behavior, sleep duration, and depression. By doing so, we aimed to enhance our understanding of these dynamics and contemplate strategies for managing and preventing depression at both the individual and societal levels. This study used a four-year dataset from the Korea National Health and Nutrition Examination Survey (KNHANES), which is notable for its representative sample of participants.

## Results

### Descriptive characteristics

This study included 21,440 participants (9,290 men and 12,150 women). Among men, 357 (4.8%) reported depressive symptoms, while 866 (7.12%) reported depressive symptoms among women. Regarding drinking habits in the past year, 81 (4.8%) of men who abstained from alcohol in the past year had depressive symptoms, while 371 (8.4%) of women reported depressive symptoms. Additionally, 132 (3.0%) men who consumed alcohol 1–4 times per month experienced depressive symptoms. Concerning the amount of alcohol consumed per occasion among men, depressive symptoms were reported in 127 (3.9%) and 79 (2.7%), among those consuming 1–2 drinks or less and 3–6 drinks, respectively. Among those who consumed ≥ 7 drinks per occasion, 4.8% had depressive symptoms. Among women, 584 (6.8%) consuming 1-2 drinks or less reported depressive symptoms, while 158 (6.2) experiencing depressive symptoms consumed 3-6 drinks per occassion. Additionally, 124 (12.9%) with depressive symptoms consumed 7 drinks or more. In terms of age at alcohol initiation among men, 235 (4.2%) who started at 19 years or younger had depressive symptoms, with a significant age-related difference (p = 0.024). Regarding sleep duration, 4.9% of  men  with below-average sleep, 3.0% with average sleep, and 3.8% with above-average sleep experienced depressive symptoms. Continuing with sleep duration, 10.3% of women below-average sleep, 4.6% with average sleep, and 6.2% with above-average sleep experienced depressive symptoms. The detailed information regarding other sociodemographic characteristics and variables can be found in Table 1.

**Table 1:** Depressive symptoms and sociodemographic characteristics of study participants.

	Study participants (n = 21,440)									
Variables	Male (n = 9,290)					Female (n = 12,150)				
	Non-depressive (n = 8,933)		Depressive (n = 357)		*P*	Non-depressive (n = 11,284)		Depressive (n = 866)		*p*
	N	%	n	%		n	%	n	%	
Past-year drinking frequency										
Have not drunk at all	1,603	95.2	81	4.8	**0.001**	4,040	91.6	371	8.4	** < 0.001**
1–4 times a month	4,210	97.0	132	3.0		5,965	94.1	375	5.9	
Twice a week or more	3,120	95.6	144	4.4		1,279	91.4	120	8.6	
Amount of alcohol consumed per occasion										
1–2 drinks or less	3,109	96.1	127	3.9	** < 0.001**	8,059	93.2	584	6.8	** < 0.001**
3–6 drinks	2,811	97.3	79	2.7		2,386	93.8	158	6.2	
7 drinks or more	3,013	95.2	151	4.8		839	87.1	124	12.9	
Age of alcohol initiation										
19 or below	5,352	95.8	235	4.2	**0.024**	3,584	92.6	288	7.4	0.365
20 or above	3,581	96.7	122	3.3		7,700	93.0	578	7.0	
Sleep duration										
Below Average	2,942	95.1	152	4.9	** < 0.001**	3,889	89.7	448	10.3	** < 0.001**
Average (6–7)	3,540	97.0	108	3.0		4,125	95.4	201	4.6	
Above average	2,451	96.2	97	3.8		3,270	93.8	217	6.2	
Age										
20–39	2,625	95.4	127	4.6	**0.019**	3,190	92.3	266	7.7	** < 0.001**
40–59	3,266	96.8	109	3.2		4,400	95.1	229	4.9	
60 or above	3,154	96.2	123	3.8		3,813	91.0	376	9.0	
Educational attainment										
Middle school or less	2,140	94.7	119	5.3	** < 0.001**	3,806	89.8	432	10.2	** < 0.001**
High school	3,127	96.1	126	3.9		3,546	93.5	247	6.5	
University or more	3,666	97.0	112	3.0		3,932	95.5	187	4.5	
Household income										
Q1 (high)	2,806	98.0	58	2.0	** < 0.001**	3,330	96.8	110	3.2	** < 0.001**
Q2	2,620	97.2	76	2.8		3,124	93.8	208	6.2	
Q3	2,136	96.2	84	3.8		2,793	92.9	214	7.1	
Q4 (low)	1,371	90.8	139	9.2		2,037	85.9	334	14.1	
Marital status										
Married	7,163	96.6	249	3.4	** < 0.001**	9,772	93.3	706	6.7	** < 0.001**
Not married	1,770	94.2	108	5.8		1,532	90.5	160	9.5	
Region										
Urban or suburban	7,182	96.1	290	3.9	0.692	9,271	93.3	669	6.7	** < 0.001**
Rural	1,751	96.3	67	3.7		2,013	91.1	197	8.9	
Employment status										
Employed	6,567	97.4	178	2.6	** < 0.001**	5,937	94.9	318	5.1	** < 0.001**
Not employed	2,366	93.0	179	7.0		5,347	90.7	548	9.3	
Smoking experience										
Non-smoker	2,162	97.5	56	2.5	** < 0.001**	10,248	94.1	640	5.9	** < 0.001**
Current smoker	3,106	94.5	182	5.5		515	78.7	139	21.3	
Ex-smoker	3,777	96.9	121	3.1		640	87.4	92	12.6	
BMI										
Underweight	207	91.2	20	8.8	** < 0.001**	541	90.8	55	9.2	**0.003**
Normal	2,691	95.8	118	4.2		5,004	93.4	351	6.6	
Overweight	2,321	96.9	74	3.1		2,373	93.6	163	6.4	
Obese	3,714	96.2	145	3.8		3,366	91.9	297	8.1	

*BMI* body mass index.

Significant values are in bold, indicating significance at p < 0.05, p < 0.01, or p < 0.001 levels.

### Association between drinking behaviors and depressive symptoms

Multivariable logistic regression analyses were performed to assess the factors associated with depressive symptoms among the study participants, with a binary dependent variable representing 0 for non-depressive symptoms and 1 for depressive symptoms.

In Table 2, men who reported drinking 1–4 times a month had significantly lower odds of experiencing depressive symptoms [odds ratio (OR) 0.64, 95% confidence interval (CI) 0.43–0.94, p = 0.024] than those who had not consumed alcohol in the past year. Similarly, women who consumed alcohol 1–4 times a month had significantly reduced odds of depressive symptoms (OR 0.73, 95% CI 0.61–0.88, p = 0.001) than those of their counterparts who abstained from alcohol. Furthermore, women who consumed ≥ 7 drinks per occasion had significantly higher odds of experiencing depressive symptoms (OR 1.63, 95% CI 1.22–2.16, p = 0.001) than those who typically consumed ≤ 1 to 2 drinks. Men who began consuming alcohol before the age of 19 had a higher likelihood of experiencing depressive symptoms (OR 1.29, 95% CI 1.00–1.66, p = 0.048).

**Table 2:** Association between drinking behaviors and depressive symptoms among study participants.

Covariates	Study participants (n = 21,440)							
	Males (n = 9290)				Females (n = 12,150)			
	OR	95% CI		*p*	OR	95% CI		*p*
Past-year drinking frequency								
Have not drunk at all	1.00				1.00			
1–4 times a month	**0.64**	**0.43**	**0.94**	**0.024**	**0.73**	**0.61**	**0.88**	**0.001**
Twice a week or more	0.89	0.58	1.38	0.612	0.78	0.59	1.04	0.087
Amount of alcohol consumed per occasion								
1–2 drinks or less	1.00				1.00			
3–6 drinks	0.86	0.58	1.26	0.427	1.09	0.87	1.37	0.448
7 drinks or more	1.25	0.86	1.83	0.240	**1.63**	**1.22**	**2.16**	**0.001**
Age of alcohol initiation								
19 or below	**1.29**	**1.00**	**1.66**	**0.048**	1.03	0.84	1.25	0.804
20 or above	1.00				1.00			

The results presented above are adjusted for sleep duration, demographic variables, and health-related variables.

*CI* confidence interval, *OR* odds ratio.

Significant values are in bold, indicating significance at p < 0.05, p < 0.01, or p < 0.001 levels.

### Association between sleep duration, covariates, and depressive symptoms

Men who reported sleeping less than 6 h had a notably increased risk of experiencing depressive symptoms (OR 1.48, 95% CI 1.14–1.92, p = 0.003) (Table 3). Conversely, individuals aged ≥ 60 had significantly lower odds of experiencing depressive symptoms. Household income played a role, with those in the lowest income quartile (Q4) and those who were unemployed showing higher odds of depressive symptoms. Smoking history, particularly among current smokers and ex-smokers, was linked to a significantly greater likelihood of depressive symptoms, as was a low body mass index (BMI) (below 18.5).

**Table 3:** Association between covariates and depressive symptoms among study participants.

Covariates	Study participants (n = 21,440)								
	Males (n = 9,290)					Females (n = 12,150)			
	OR		95% CI		*p*	OR	95% CI		*P*
Sleep duration									
Below average	**1.48**	**1.14**		**1.92**	**0.003**	**2.13**	**1.78**	**2.54**	** < 0.001**
Average	1.00					1.00			
Above average	1.07	0.80		1.42	0.655	1.22	1.00	1.50	0.051
Age									
20–39	1.00					1.00			
40–59	0.74	0.53		1.02	0.067	**0.75**	**0.59**	**0.95**	**0.020**
60 or above	**0.42**	**0.27**		**0.65**	** < 0.001**	**0.69**	**0.51**	**0.93**	**0.015**
Educational attainment									
Middle school or less	1.29	0.92		1.81	0.143	**1.92**	**1.48**	**2.50**	** < 0.001**
High school	0.92	0.70		1.21	0.564	1.23	1.00	1.52	0.053
University or more	1.00					1.00			
Household income									
Q1 (high)	1.00					1.00			
Q2	1.36	0.96		1.93	0.085	**1.76**	**1.38**	**2.25**	** < 0.001**
Q3	**1.79**	**1.26**		**2.55**	**0.001**	**1.80**	**1.41**	**2.30**	** < 0.001**
Q4 (low)	**4.23**	**2.94**		**6.10**	** < 0.001**	**3.22**	**2.50**	**4.15**	** < 0.001**
Marital status									
Married	1.00					1.00			
Not married	1.18	0.85		1.65	0.332	**1.62**	**1.27**	**2.06**	** < 0.001**
Region									
Urban or suburban	1.00					1.00			
Rural	0.85	0.64		1.13	0.271	1.17	0.98	1.40	0.077
Employment status									
Employed	1.00					1.00			
Not employed	**2.38**	**1.83**		**3.09**	** < 0.001**	**1.64**	**1.41**	**1.92**	** < 0.001**
Smoking experience									
Non-smoker	1.00					1.00			
Current smoker	**2.16**	**1.56**		**3.01**	** < 0.001**	**3.51**	**2.77**	**4.44**	** < 0.001**
Ex-smoker	1.29	0.91		1.81	0.152	**2.19**	**1.70**	**2.81**	** < 0.001**
BMI									
Underweight	**1.73**	**1.04**		**2.89**	**0.035**	1.26	0.92	1.73	0.145
Normal	1.00					1.00			
Overweight	0.90	0.66		1.22	0.495	0.90	0.74	1.10	0.315
Obese	1.04	0.81		1.35	0.746	1.01	0.85	1.20	0.877

The results presented above are adjusted for alcohol-related variables, including drinking frequency, amount consumed per occasion, and age of alcohol initiation.

*BMI* body mass index, *CI* confidence interval, *OR* odds ratio.

Significant values are in bold, indicating significance at p < 0.05, p < 0.01, or p < 0.001 levels.

Women with inadequate sleep duration (˂ 6 h) had a higher risk (OR 2.13, 95% CI 1.78–2.54, p < 0.001), whereas women aged 40–59 and ≥ 60 years had notably lower odds of experiencing depressive symptoms. Educational attainment played a role, with those who had completed middle school or less showing an increased risk than those with university-level education. Household income, especially in the higher quartiles (Q2, Q3, and Q4), marital status (unmarried), and employment status (unemployed) were all significant factors associated with a higher likelihood of depressive symptoms. Furthermore, smoking experience, including both current and ex-smokers, was significantly associated with a greater risk of depressive symptoms than non-smoking.

### Association between drinking behaviors and depressive symptoms among men by sleep duration

Based on the sleep duration, men were categorized into three distinct groups: "below average," "average," and "above average," each with unique characteristics and associated risk factors, and a subgroup analysis was performed (Table 4).

**Table 4:** Sleep duration and the association between drinking behaviors and depressive symptoms among men (n = 9290).

Covariates	Below average (less than 6) (n = 3094)				Average (6–7 h) (n = 3648)				Above average (8 h or more) (n = 2548)			
	OR	95% CI		*P*	OR	95% CI		*p*	OR	95% CI		*p*
**Past-year drinking frequency**												
Have not drunk at all	1.00				1.00				1.00			
1–4 times a month	**0.44**	**0.23**	**0.84**	**0.013**	0.79	0.39	1.60	0.509	0.87	0.43	1.74	0.684
Twice a week or more	0.55	0.27	1.13	0.101	1.24	0.57	2.70	0.585	1.19	0.55	2.59	0.664
Amount of alcohol consumed per occasion												
1–2 drinks or less	1.00				1.00				1.00			
3–6 drinks	1.09	0.57	2.09	0.787	0.76	0.40	1.46	0.412	0.77	0.37	1.58	0.474
7 drinks or more	1.61	0.85	3.05	0.145	0.89	0.47	1.69	0.726	1.49	0.74	2.98	0.261
Age of alcohol initiation												
19 or below	1.22	0.82	1.82	0.327	1.52	0.95	2.42	0.082	1.19	0.74	1.92	0.472
20 or above	1.00				1.00				1.00			
Age												
20–39	1.00				1.00				1.00			
40–59	**0.59**	**0.36**	**0.95**	**0.032**	**0.53**	**0.30**	**0.94**	**0.031**	1.70	0.82	3.50	0.152
60 or above	**0.28**	**0.14**	**0.53**	** < 0.001**	**0.29**	**0.19**	**0.86**	**0.019**	1.02	0.40	2.59	0.963
Educational attainment												
Middle school or less	1.38	0.81	2.34	0.232	1.20	0.63	2.26	0.583	1.31	0.73	2.41	0.439
High school	0.82	0.53	1.27	0.368	0.81	0.51	1.30	0.381	2.51	1.04	6.08	0.349
University or more	1.00				1.00				1.00			
Household income												
Q1 (high)	1.00				1.00				1.00			
Q2	0.95	0.56	1.63	0.860	1.55	0.88	2.74	0.132	**2.51**	**1.04**	**6.08**	**0.041**
Q3	1.48	0.87	2.52	0.144	1.65	0.91	3.01	0.102	**3.48**	**1.46**	**8.30**	**0.005**
Q4 (low)	**4.09**	**2.33**	**7.18**	** < 0.001**	**4.20**	**2.22**	**7.93**	** < 0.001**	**6.01**	**2.51**	**14.39**	** < 0.001**
Marital status												
Married	1.00				1.00				1.00			
Not married	1.20	0.74	1.95	0.468	1.04	0.57	1.89	0.893	1.64	0.80	3.38	0.178
Region												
Urban or suburban	1.00				1.00				1.00			
Rural	0.65	0.40	1.05	0.080	0.97	0.58	1.62	0.911	1.07	0.65	1.77	0.788
Employment status												
Employed	1.00				1.00				1.00			
Not employed	**1.97**	**1.29**	**2.99**	**0.002**	**2.00**	**1.24**	**3.21**	**0.004**	**3.93**	**2.35**	**6.58**	** < 0.001**
Smoking experience												
Non-smoker	1.00				1.00				1.00			
Current smoker	**2.41**	**1.45**	**4.01**	**0.001**	**2.69**	**1.42**	**5.08**	**0.002**	1.44	0.77	2.68	0.256
Ex-smoker	1.02	0.58	1.79	0.954	**2.10**	**1.10**	**4.00**	**0.024**	0.98	0.54	1.80	0.950
BMI												
Underweight	**3.23**	**1.66**	**6.28**	**0.001**	1.00	0.29	3.38	0.994	0.64	0.14	2.81	0.550
Normal	1.00				1.00				1.00			
Overweight	0.91	0.56	1.49	0.720	0.91	0.54	1.54	0.732	0.83	0.46	1.50	0.542
Obese	1.11	0.73	1.68	0.631	0.88	0.55	1.40	0.586	1.18	0.73	1.92	0.498

*BMI* body mass index, *CI* confidence interval, *OR* odds ratio.

Significant values are in bold, indicating significance at p < 0.05, p < 0.01, or p < 0.001 levels.

Men in the "below average" sleep duration group, characterized by ˂ 6 h of sleep, showed several noteworthy findings. Those who reported drinking 1–4 times a month had significantly lower odds of experiencing depressive symptoms (OR 0.44, 95% CI 0.23–0.84, p = 0.013) than those who had not consumed alcohol in the past year. Additionally, men aged 40–59 and ≥ 60 years exhibited lower odds of depressive symptoms than those in the reference group (range: 20–39). Furthermore, household income and employment status were significant factors, with the lowest income quartile (Q4) and unemployment associated with higher odds of depressive symptoms. Moreover, smoking and a low BMI were linked to increased odds of developing depressive symptoms in this group.

Among men in the "average" sleep duration group (6–7 h), age, household income, employment status, smoking experience, and BMI were significant factors. Older men aged between 40 and 59 and above 60 years displayed lower odds of depressive symptoms than those in the reference group (age range: 20–39). Similarly, in the lowest income quartile (Q4), the unemployed, current smokers, and ex-smokers exhibited higher odds of depressive symptoms.

Household income and employment status were significant factors for men in the "above average" sleep duration group (≥ 8 h). Specifically, men in the lowest income quartile (Q4) had substantially higher odds of experiencing depressive symptoms than those in the highest income quartile (Q1). Not being employed was also associated with increased odds of depressive symptoms in this group.

### Association between drinking behaviors and depressive symptoms among women by sleep duration

Table 5 outlines the findings derived from the logistic regression analysis and summarizes the factors influencing the probability of depressive symptoms among women stratified by their sleep duration.

**Table 5:** Sleep duration and the association between drinking behaviors and depressive symptoms among women (n = 12,150).

Covariates	Below average (less than 6) (n = 4337)				Average (6–7 h) (n = 4326)				Above average (8 h or more) (n = 3487)			
	OR	95% CI		*P*	OR	95% CI		*p*	OR	95% CI		*P*
Past-year drinking frequency												
Have not drunk at all	1.00				1.00				1.00			
1–4 times a month	**0.73**	**0.56**	**0.95**	**0.018**	0.71	0.48	1.06	0.091	0.75	0.52	1.08	0.119
Twice a week or more	0.89	0.60	1.32	0.560	**0.39**	**0.21**	**0.73**	**0.003**	1.12	0.64	1.93	0.697
Amount of alcohol consumed per occasion												
1–2 drinks or less	1.00				1.00				1.00			
3–6 drinks	0.89	0.60	1.32	0.727	**1.67**	**1.08**	**2.57**	**0.021**	0.67	0.40	1.11	0.118
7 drinks or more	**1.06**	**0.77**	**1.47**	**0.043**	**1.83**	**1.05**	**3.17**	**0.032**	1.58	0.93	2.70	0.092
Age of alcohol initiation												
19 or below	1.06	0.79	1.42	0.701	1.18	0.80	1.72	0.409	0.86	0.57	1.30	0.474
20 or above	1.00				1.00				1.00			
Age												
20–39	1.00				1.00				1.00			
40–59	0.80	0.56	1.15	0.229	**0.56**	**0.35**	**0.91**	**0.019**	0.87	0.53	1.40	0.554
60 or above	0.81	0.52	1.25	0.334	**0.44**	**0.24**	**0.81**	**0.009**	0.73	0.39	1.36	0.319
Educational attainment												
Middle school or less	**1.76**	**1.21**	**2.55**	**0.003**	**2.40**	**1.43**	**4.03**	**0.001**	**2.02**	**1.19**	**3.45**	**0.010**
High school	1.24	0.92	1.69	0.165	1.20	0.80	1.80	0.385	1.24	0.82	1.89	0.305
University or more	1.00				1.00				1.00			
Household income												
Q1 (high)	1.00				1.00				1.00			
Q2	1.25	0.88	1.76	0.208	**2.60**	**1.61**	**4.19**	** < 0.001**	**2.39**	**1.44**	**3.98**	**0.001**
Q3	**1.53**	**1.09**	**2.16**	**0.014**	**2.18**	**1.32**	**3.62**	**0.003**	**2.11**	**1.25**	**3.54**	**0.005**
Q4 (low)	**2.89**	**2.04**	**4.09**	** < 0.001**	**3.48**	**2.04**	**5.95**	** < 0.001**	**3.64**	**2.13**	**6.22**	** < 0.001**
Marital status												
Married	1.00				1.00				1.00			
Not married	**1.83**	**1.28**	**2.61**	**0.001**	1.32	0.82	2.13	0.254	1.48	0.92	2.36	0.106
Region												
Urban or suburban	1.00				1.00				1.00			
Rural	0.95	0.73	1.25	0.733	**1.56**	**1.10**	**2.19**	**0.012**	1.28	0.92	1.78	0.142
Employment status												
Employed	1.00				1.00				1.00			
Not employed	**1.70**	**1.36**	**2.12**	** < .001**	**1.64**	**1.20**	**2.23**	**0.002**	**1.50**	**1.11**	**2.05**	**0.010**
Smoking experience												
Non-smoker	1.00				1.00				1.00			
Current smoker	**2.94**	**2.07**	**4.19**	** < 0.001**	**3.64**	**2.31**	**5.75**	** < 0.001**	**4.92**	**3.11**	**7.79**	** < 0.001**
Ex-smoker	**2.00**	**1.38**	**2.90**	** < 0.001**	**2.05**	**1.24**	**3.38**	**0.005**	**2.96**	**1.84**	**4.75**	** < 0.001**
BMI												
Underweight	1.34	0.88	2.05	0.178	1.44	0.76	2.71	0.265	0.91	0.43	1.89	
Normal	1.00				1.00				1.00			0.791
Overweight	0.82	0.62	1.09	0.174	1.15	0.77	1.72	0.510	0.86	0.57	1.29	0.467
Obese	0.89	0.70	1.14	0.369	1.17	0.83	1.66	0.373	1.17	0.84	1.64	0.360

*BMI* body mass index, *CI* confidence interval, *OR* odds ratio.

Significant values are in bold, indicating significance at p < 0.05, p < 0.01, or p < 0.001 levels.

Several significant factors are implicated in the "below average" sleep duration group, which comprises individuals with ˂6 h of sleep. Notably, individuals in this category who reported consuming alcohol 1–4 times a month displayed significantly reduced odds of experiencing depressive symptoms than those who refrained from alcohol (OR 0.73, 95% CI 0.56–0.95, p = 0.018). Additionally, the quantity of alcohol consumed per occasion played a role, with those who consumed ≥ 7 drinks demonstrating increased odds of depressive symptoms (OR 1.06, 95% CI 0.77–1.47, p = 0.043) than those in the reference group (> 1–2 drinks). Socioeconomic factors, including lower educational attainment, lower household income, marital status, unemployment, and smoking history, were associated with elevated odds of depressive symptoms within this group.

Both drinking frequency and the amount of alcohol consumed per occasion emerged as significant factors in the "average" sleep duration group (6–7 h). Individuals who consumed alcohol twice a week or more exhibited lower odds of experiencing depressive symptoms (OR 0.39, 95% CI 0.21–0.73, p = 0.003). However, in terms of alcohol quantity, those who consumed 3–6 drinks (OR 1.67, 95% CI 1.08–2.57, p = 0.021) and ≥ 7 drinks (OR 1.83, 95% CI 1.05–3.17, p = 0.032) had significantly higher odds of depressive symptoms. Age, educational level, household income, employment status, and smoking history were significant factors. Older women exhibited lower odds of experiencing depressive symptoms than did their younger counterparts (aged 20–39 years). A lower educational level (middle school or lower) was associated with higher odds of developing depressive symptoms. Household income played a crucial role, with individuals in the second, third, and fourth income quartiles experiencing substantially higher odds of experiencing depressive symptoms. Additionally, living in a rural area, unemployment, current smoking, and past smoking were all linked to an increased likelihood of depressive symptoms within this category.

No significant association between alcohol-related variables and depressive symptoms was observed in the "above average" sleep duration group (≥ 8 h). However, educational attainment, household income, employment status, and smoking history played a significant role. Lower educational attainment was associated with higher odds of experiencing depressive symptoms. Individuals in the second, third, and fourth income quartiles had notably elevated odds of depressive symptoms compared with those in the highest income quartile (Q1). Moreover, not being employed was associated with an increased likelihood of depressive symptoms in this group. Individuals who were current smokers or had a history of smoking had greater odds of experiencing depressive symptoms.

## Discussion

### Research findings

In our study, we delved into the intricate interplay between alcohol consumption, sleep duration, and depressive symptoms in a diverse participant cohort. Both men and women who consumed alcohol 1–4 times a month had significantly lower odds of depressive symptoms than those who abstained. However, women who consumed ≥ 7 drinks per occasion had higher odds of depressive symptoms. Men who started consuming alcohol before the legal drinking age of 19, which is the legal age in Korea, had increased odds of experiencing depressive symptoms compared with those who began drinking at the legal age. Additionally, factors such as sleep duration, age, educational attainment, income, employment status, marital status, smoking history, and BMI were significantly associated with depressive symptoms in the different sleep duration and sex subgroups. These findings provide valuable insights into the complex interplay between drinking behavior, sleep duration, and depressive symptoms among men and women.

Our study underscores the inherent complexity of the relationship between alcohol consumption and depressive symptoms. Particularly noteworthy was the protective effect associated with moderate drinking frequency (1–4 times a month) against the development of depressive symptoms in both sexes.

However, the situation becomes more nuanced, especially for women, for whom heavy alcohol consumption per occasion (≥ 7 drinks per occasion) is associated with an elevated risk of depressive symptoms. These findings emphasize the importance of considering not only how often but also how much alcohol is consumed, particularly among women. Our study revealed that women who reported consuming seven or more alcoholic beverages per occasion had a greater likelihood of experiencing depressive symptoms than their male counterparts.

Furthermore, our study underscores the profound effect of sleep on depressive symptoms in both sexes. In particular, individuals with below-average sleep duration exhibited significantly elevated odds of experiencing depressive symptoms. This finding highlights the critical importance of prioritizing healthy sleep habits as a key factor in reducing the risk of depressive symptoms in individuals of all sexes.

### Drinking behaviors, sleep duration, and depressive symptoms

Particularly noteworthy was the protective effect associated with moderate alcohol consumption (1–4 times a month) against the development of depressive symptoms in both sexes. This is consistent with the findings of a previous study, which reported that abstaining from alcohol increases the likelihood of developing depression compared to light drinking^9^. Moreover, a recent longitudinal study examining the association between moderate alcohol consumption and depression showed that individuals who consistently consumed alcohol in moderate quantities, occasionally or regularly, were projected to have lower depressive symptoms and lower rates of probable depression upon attaining 50 years of age than those who consistently abstained from alcohol^14^. This suggests that our findings were consistent with those of previous studies that explored the relationship between alcohol consumption and depressive symptoms. Individuals who do not consume alcohol tend to be less socially active and have weaker social support, which could introduce potential confounding factors when investigating the relationship between alcohol consumption and the development of depression^15^. Another study highlighted that alcohol consumption is a prevailing societal norm among adults and is of substantial importance in social contexts^16^. Based on these findings, it can be inferred that alcohol consumption influences social relationships and ultimately impacts mental well-being.

Our study revealed that women who consumed seven or more alcoholic beverages per occasion had a greater likelihood of experiencing depressive symptoms than those who consumed one or two alcoholic beverages per occasion. This observation resonates with previous findings^17^, which highlighted that intoxication and alcohol consumption were linked to reduced mental well-being in women. The sex-based differences in the relationship between alcohol consumption and depression can be explained by this research finding^18^, indicating that in the case of men, alcohol consumption is used to cope with depression, whereas this is not the case for women. Therefore, heavy alcohol consumption may have a greater effect on the induction of depressive symptoms in women.

Age at alcohol consumption initiation has been a topic of interest in relation to mental health, particularly depression. Individuals who start drinking alcohol at a young age, typically during adolescence, may be at increased risk of developing suicidal ideation and depressive symptoms later in life^19^. Furthermore, our study found that men who started drinking alcohol before the legal drinking age were more likely to experience depressive symptoms, whereas this factor did not influence women. Interestingly, earlier studies did not highlight sex differences in the association between age at alcohol initiation and depressive symptoms; however, our study revealed such a sex disparity. This divergence can be attributed to cultural differences because our study focused exclusively on South Korean respondents, whereas previous research^18^ involved participants in the United States. Complex sociocultural factors might have contributed to this finding, and further investigation is necessary to uncover the underlying causes.

### Sleep duration and depressive symptoms

Sleep duration emerged as a notable factor influencing depressive symptoms even after adjusting for other variables. Expanding on prior research, our study delved into the potential role of sleep duration as a moderating variable when exploring the connection between drinking patterns and depressive symptoms. We conducted a subgroup analysis after dividing the participants based on sleep duration into the following groups: below-average, average, and above-average. Among men with ˂6 h of sleep, those who reported drinking 1–4 times a month had significantly lower odds of experiencing depressive symptoms than those who had not consumed alcohol in the past year. Men with alcohol-related issues often experience lower sleep quality, whereas moderate drinking does not have the same effect^20,21^. For this reason, individuals with insufficient sleep may not consistently exhibit depressive symptoms, particularly when their alcohol consumption remains moderate.

Among women in the "below average sleep group," those who consumed alcohol 1–4 times a month had lower odds of experiencing depressive symptoms than those who had never consumed. In terms of the amount of alcohol consumed per occasion, those who consumed ≥ 7 drinks per occasion had higher odds of depressive symptoms than those who consumed ≤ 1–2 drinks. In the "average sleep group," drinking frequency was not linked to depressive symptoms; however, based on the amount of alcohol consumed per occasion, those who consumed 3–6 drinks or ≥ 7 drinks per occasion had elevated odds of depressive symptoms. In summary, although drinking frequency did not increase the risk of depressive symptoms, the amount of alcohol consumed per occasion emerged as a risk factor, even among those with "average sleep." Binge drinking, not average intake, is a risk factor for depressive symptoms. Heavier alcohol use was also associated with depression^22,23^. These findings suggest that heavy alcohol consumption may lead to depressive symptoms. In addition, the association between problematic drinking and psychological distress was more pronounced in women than in men^24^. These results confirm the existence of sex disparities in alcohol-related psychological issues and highlight women's greater vulnerability to the adverse effects of alcohol on their mental health. These results are also consistent with those of an earlier study^25^ that highlighted the increased severity of medical, psychiatric, and functional repercussions associated with substance use disorders in women. The observed gender disparities in alcohol and substance use, as well as psychological issues, may be linked to women's coping mechanisms in response to stress and negative emotions.

### Socioeconomic, health factors and depressive symptoms

In examining the covariates and depressive symptoms, it was found that those aged 40–59 years and those aged ≥ 60 years experienced fewer depressive symptoms than those aged 20–39 years, indicating a trend of decreasing depressive symptoms with age. This study's findings are consistent with those of prior research^26,27^. As for educational attainment, women with lower education status were more likely to exhibit depressive symptoms, a pattern not identified in previous research^28^. To gain further insight, a more detailed examination of the relationship between sex, educational attainment, and depression is required. Both men and women exhibited a strong association between income levels and depressive symptoms. As income levels decreased, the likelihood of experiencing depressive symptoms increased significantly, a finding consistent with prior research^29^. Interestingly, in our study employment status, emerged as a significant factor affecting depressive symptoms, with both men and women experiencing a higher likelihood of depression when unemployed. Among women, being unmarried was associated with a greater probability of encountering depressive symptoms than their married counterparts were, aligning with previous research^30^. The smoking experience was another noteworthy factor, with different patterns between men and women. Among men, current smokers had a higher likelihood of experiencing depressive symptoms. Intriguingly, both past and current smokers were more likely to experience depressive symptoms than non-smokers. An association in a previous study also suggests that smoking has a substantial impact on women’s mental well-being.^31^

### Strengths and limitations of the study

Our study had several strengths. First, it leverages data from the KNHANES, a nationally representative dataset, allowing us to make inferences about the broader population, thereby enhancing the reliability of our findings. The substantial and diverse sample sizes bolstered the statistical power of our analyses. Second, our study not only investigated the factors associated with depressive symptoms but also controlled for potential confounding variables, enhancing the reliability and validity of our findings. We investigated the impact of sleep duration and conducted subgroup analyses to gain insight into its influence. This comprehensive approach allowed us to gain a deeper understanding of the interplay between these variables and depression. Moreover, by stratifying our analyses by sex, we unveiled sex-specific patterns in the relationships between variables, enabling more precise, sex-sensitive interventions and underscoring the importance of considering sex in mental health research.

Despite offering valuable insights, our study had certain limitations. First, its cross-sectional design precluded the establishment of causality among the examined variables. Consequently, we could not definitively determine the direction of influence or identify the temporal sequence of events. Second, despite our comprehensive examination of various factors, our study did not encompass all the potential variables contributing to depressive symptoms. Unmeasured or unaccounted for variables, both psychological and physiological, may have influenced the observed associations. Third, as with any large-scale survey, issues related to recall bias and self-reporting, particularly when assessing alcohol consumption and sleep duration, may exist. Especially concerning the key variable, drinking behavior, pertinent information regarding individuals' motives for alcohol consumption and the types of alcohol consumed was inaccessible due to data limitations. In future studies, we anticipate a comprehensive inclusion of factors that could influence drinking behavior, such as motives for alcohol consumption and types of alcohol consumed. These limitations should be considered when interpreting our findings and underscore the need for further research to address the complexities in the relationship between alcohol consumption, sleep, and depressive symptoms.

### Conclusion

Our study explored the intricate relationships between alcohol consumption, sleep duration, and depressive symptoms in a diverse participant cohort. Notably, among men, moderate alcohol consumption (1–4 times a month) was associated with significantly lower odds of depressive symptoms than abstaining; a similar pattern was observed among women. Conversely, women who consumed ≥ 7 drinks per occasion had elevated odds of experiencing depressive symptoms. Men who started consuming alcohol before the age of 19 showed higher odds of developing depressive symptoms.

In summary, the impact of depression extends far beyond individual suffering and affects various facets of life and society. The relationship between alcohol consumption, sleep duration, and depression is complex and multifaceted and requires comprehensive investigation. Our findings provide a foundation for more tailored approaches to depression prevention and treatment, with a focus on improving mental well-being. Further research is warranted to establish causality regarding the association between drinking behavior and depression with respect to sex differentiation and sleep duration, ideally involving diverse geographical regions.

## Methods

### Design and setting

**Figure 1:**
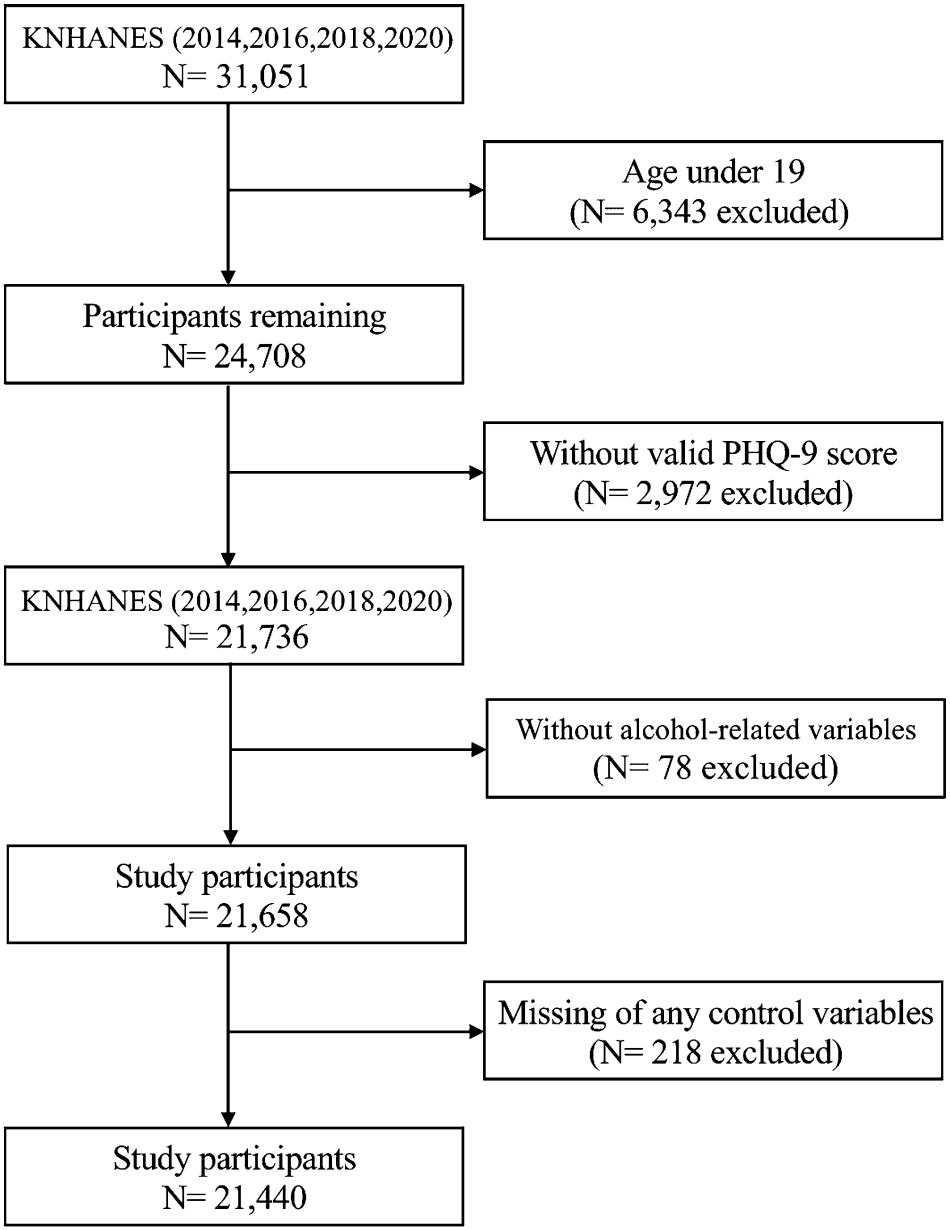
Flow diagram of the study participants. *KNHAES* Korea National Health and Nutritional Examination Survey, *PHQ-9* Patient Health Questionnaire-9.

In this study, we performed a secondary analysis of data obtained from the KNHANES in 2014, 2016, 2018, and 2020. We focused on these years because mental health indicators such as the Patient Health Questionnaire-9 (PHQ-9) were included exclusively in even-numbered years. The KNHANES is a nationwide cross-sectional survey conducted by the Korea Disease Control and Prevention Agency. Employing a comprehensive sampling approach, the survey ensured representation across various regions, sexes, age, and income groups in Korea, a methodology maintained since 1998. The primary purpose was to shape and assess health policies in South Korea. The KNHANES involves a combination of health assessments, health interviews, and nutrition surveys. Among the total 31,051 respondents from the four survey years, 24,708 participants aged ≥ 20 years met the inclusion criteria. After excluding individuals with incomplete data, an analysis was conducted on a sample of 21,440 respondents (Fig. 1).

All participants provided written informed consent, and the study adhered to the ethical principles of the Declaration of Helsinki. This research was not subject to institutional review board approval as it utilized publicly accessible secondary data in accordance with governmental regulations. Detailed information on the monitoring process is available to the public and can be obtained from the official KNHANES website (http://knhanes.kdca.go.kr/).

### Clinical variables

#### Depressive symptoms

We used the PHQ-9 as a quantitative tool to screen for depressive symptoms. This instrument aligns with the nine criteria outlined in the Diagnostic and Statistical Manual of Mental Disorders IV for major depressive disorder. Each criterion was rated on a scale of 0–3, with the total score ranging from 0 to 27. The PHQ-9 scores have been collected from the KNHANES biennially since 2014. This questionnaire is a valid tool for assessing the severity of depressive symptoms. A previous validation study demonstrated 88% sensitivity and 88% specificity using a cutoff score of 10^32^. Another validation study conducted in Korea^33^ reported that when considering the criterion for setting the optimal cutoff point for discriminating Major Depressive Disorder (MDD) with a preference for higher sensitivity over specificity and aiming for a low rate of false positives, this study suggests that the optimal cutoff point for MDD discrimination should be set at 10 points or higher (with a sensitivity of 86.5% and a specificity of 52.9%). Based on previous studies, individuals with significant depressive symptoms were identified using a PHQ-9 score of 10 or higher. Therefore, we categorized depressive symptom levels into two groups: the non-depressive (scores 0–9) and the depressive (scores ≥ 10).

#### Drinking behaviors and sleep duration

The primary factors examined as independent variables in this study were related to drinking behavior. Specifically, the study focused on three aspects: the frequency of alcohol consumption over the previous year, quantity of alcohol consumed per occasion, and age at which individuals first began consuming alcohol.

To evaluate how often participants had consumed alcohol in the past year, they were asked about their drinking frequency. We categorized this frequency into three groups: those who had never consumed alcohol, those who consumed 1–4 times a month, and those who consumed twice a week or more. Additionally, we determined the amount of alcohol consumed based on the number of drinks per drinking occasion, classifying it into three categories: 1–2 drinks or less, 3–6 drinks, and ≥ 7 drinks. Additionally, the study included inquiries about the age at which individuals first began drinking alcohol, categorizing them into two groups: those who began drinking at 19 years of age or earlier, and those who began drinking at 20 years of age or later. This differentiation aligns with the legal drinking age in Korea, which is set at 19 years.

Another significant independent variable in the study pertained to the hours of sleep. Respondents were asked about the number of hours they typically sleep on weekdays. We categorized the participants into three groups based on their sleep duration, which was centered around an average of 6–7 h. Those with ˂ 6 h of sleep were classified as the "below-average group", individuals sleeping around the average hours were categorized as "average", and those who slept for ≥ 8 h were designated as the "above-average group".

### Covariates

The demographic and socioeconomic factors included sex, age, educational attainment, household income, marital status, residential area, and employment status. The KNHANES participants identified themselves as male or female, and their reported sex designations were adopted. Based on age, participants were categorized into three groups: 20–39, 40–59, and ≥ 60. Educational attainment was determined by the type of diploma. Participants were grouped into three categories based on their educational attainment. Regarding household income, the participants were divided into quartiles, with Q1 representing the highest income group and Q4 the lowest. We categorized marital status by including respondents who reported being married and excluding those who were divorced or separated from the "not married" category. This approach allows for clearer differentiation between individuals who are currently in a marital union and those who are not. Occupational status was categorized based on employment status. Those who reported being employed, whether on a full-time or part-time basis, were classified as "employed". Conversely, those who indicated that they were not employed were categorized as "not employed".

Health-related variables included smoking history and body mass index (BMI). Participants were categorized into three groups: individuals who used to smoke (ex-smokers), those currently smoking (current smokers), and those who have never smoked (non-smokers). According to the Asian BMI classification^34^, BMI was classified into four groups: underweight (< 18.5), normal weight (18.5–23), overweight (23–25), and obesity (≥ 25).

### Statistical analysis

All statistical analyses were performed using SAS version 9.4 (SAS Institute, Cary, North Carolina, USA). Chi-squared tests were used to evaluate variations in categorical variables concerning the presence of notable depressive symptoms. We investigated the elements linked to significant depressive symptoms in each sex separately. To examine the relationship between variables such as alcohol use, sociodemographic factors, health-related aspects, and depressive symptoms, we conducted a multivariable logistic regression analysis. Furthermore, we conducted a subgroup analysis to assess potential variations in the variables based on sleep duration. Our calculations produced ORs with corresponding 95% CIs, and a p-value of less than 0.05 indicated statistical significance.

## Data Availability

The KNHANES database is available to the public and can be found at the following link: https://knhanes.kdca.go.kr.
